# Identification of species in the genus Nitraria L. (Nitrariaceae) based on nucleotide variability of nuclear ribosomal DNA

**DOI:** 10.18699/VJ20.640

**Published:** 2020-08

**Authors:** Т.А. Poliakova, E.V. Banaev, M.A. Tomoshevich

**Affiliations:** Vavilov Institute of General Genetics of the Russian Academy of Sciences, Moscow, Russia; Central Siberian Botanical Garden of Siberian Branch of the Russian Academy of Sciences, Novosibirsk, Russia; Central Siberian Botanical Garden of Siberian Branch of the Russian Academy of Sciences, Novosibirsk, Russia

**Keywords:** Nitraria, N. schoberi, N. sibirica, N. komarovii, genetic variability, taxonomy, molecular identification, ITS, transition, Nitraria, N. schoberi, N. sibirica, N. komarovii, генетическая изменчивость, таксономия, молекулярная идентификация, ITS, транзиция

## Abstract

Intragenomic polymorphism of ITS1 and ITS2 of nuclear ribosomal DNA sequences was analysed in 33 samples belonging to the Nitraria species N. schoberi, N. sibirica, and N. komarovii. The nucleotide variability of the ITS region was detected in the Nitraria species as single-nucleotide substitutions (mainly transitions) and single-nucleotide deletion. Information about the nucleotide variability of fragments is given for the first time by us. The ITS1-5.8S-ITS2 region contained 17 phylogenetically informative single-nucleotide polymorphisms. Eleven single-nucleotide substitutions (transitions, C/T) were detected in ITS1. The ITS2 spacer contained 273–274 bp and was more conservative. A total of 5 phylogenetically informative single-nucleotide polymorphisms (4 transitions: C/T, G/A, one transversion: G/C), one single-nucleotide deletion (T/–) were detected in ITS2. The average GC content was 61.5 %. The GC content was lower in N. sibirica (59.2 %) than in N. schoberi and N. komarovii (62.7 %). It has been shown that the shorter ITS2 is a suitable molecular marker separating these species, due to the low interspecific variability and simultaneous available intraspecific variability. Phylogenetic ML and BI trees constructed separately for the ITS1 and ITS2 spacers, as well as separately for the full-size ITS region and the ITS2 spacer, were congruent. The results obtained on the intraspecific differentiation of N. sibirica revealed two main ribotypes among the samples of this species: the main Siberian sibirica-ribotype and the main Kazakh sibiricaribotype. Geographical features of the distribution of N. sibirica ribotypes, as well as the presence of significant differences between the main Siberian and Kazakh sibirica-ribotypes (3 single-nucleotide substitutions) indicated significant inter-population differences and taxonomic heterogeneity of N. sibirica. Most likely, the processes of homogenization of nuclear ribosomal DNA of N. sibirica samples, the origin of which is associated with hybridization and speciation, are currently continuing.

## Abstract (ru)

Проведен сравнительный анализ внутригеномного полиморфизма последовательностей вну- тренних транскрибируемых спейсеров ITS1 и ITS2 ядерной рибосомной ДНК у 33 образцов, принадлежащих трем видам Nitraria – N. schoberi, N. sibirica, N. komarovii. Выявлена нуклеотидная изменчивость региона ITS у изученных видов Nitraria в виде однонуклеотидных замен (преимущественно транзиции) и однонуклеотидной делеции. Сведения о нуклеотидной изменчивости фрагментов приводятся впервые нами. Регион ITS1-5.8S-ITS2 у изученных видов Nitraria содержит 17 филогенетически информативных однонуклеотидных замен. В межгенном спейсере ITS1 выявлено 11 однонуклеотидных замен – транзиций (C/T). Спейсер ITS2 содержит 273–274 п. н. и отличается большей консервативностью. Всего в ITS2 у изученных образцов вы- явлено пять филогенетически информативных однонуклеотидных замен (четыре транзиции: C/T, G/A, одна трансверсия: G/C), одна однонуклеотидная делеция (T/–). Среднее значение содержания G+C составляет 61.5 %. Величина содержания GC-состава ниже у N. sibirica (59.2 %), чем у N. schoberi и N. komarovii (62.7 %). В сравнении с полноразмерным фрагментом ITS, более короткий ITS2 является подходящим молекулярным маркером, дискриминирующим виды, из-за низкой межвидовой изменчивости и одновременно выражен- ной внутривидовой вариабельности. Филогенетические ML и BI деревья, построенные как отдельно по спейсерам ITS1 и ITS2, так и отдельно по полноразмерному ITS-региону и спейсеру ITS2, оказались конгру- энтны. Полученные результаты по внутривидовой дифференциации N. sibirica позволяют выделить среди образцов этого вида два основных риботипа: основной сибирский sibirica-риботип и основной казахстан- ский sibirica-риботип. Географические особенности распространения риботипов N. sibirica, а также наличие существенных различий между основными сибирским и казахстанским sibirica-риботипами (три однонуклеотидные замены) свидетельствуют о существенных межпопуляционных различиях и таксономической неоднородности N. sibirica. Вероятнее всего, в настоящее время продолжаются процессы гомогенизации рибосомной ДНК образцов N. sibirica, происхождение которых связано с гибридизацией и видообразова- нием.

## Introduction

The molecular approach is now becoming a common aspect of plant research at the various taxonomic levels. Non-encoded regions of internal transcribed spacers (ITS) nuclear ribosomal DNA genes are the most promising molecular markers for plant taxa identification (CBOL, 2009; Shneyer, Rodionov, 2018). Along with other DNA fragments, ITS1 and ITS2 spacers were recognized as standard DNA barcodes (Hollingsworth, 2011; Li et al., 2011; Shneyer, Rodionov, 2018). Correct identification of plant species is established in 80 % of cases using only ITS marker, which is significantly higher than the commonly used loci in plant DNA barcoding (Bolson et al., 2015). Despite the limitations of ITS region, which consist in the presence of several thousand copies of sequences at the same time, including those located on different chromosomes (Song et al., 2012; Rodionov et al., 2016), the ITS locus was recognized as the most significant in the molecular taxonomy research of closely related taxa. The high importance of the entire ITS region in plant species identification was shown, for example, for genus Spiraea (Polyakova et al., 2015), Uncaria (Zhang et al., 2015), Artemisia (Wang et al., 2016). The high significance of ITS2 spacer in plant species identification was also revealed (Gao et al., 2010; Ren et al., 2010; Zhang et al., 2015; Feng et al., 2016). First of all, the success of using ITS spacers is related to efficient amplification, optimal size of amplicons for sequencing, and the level of divergence acceptable for interspecies comparisons (Shneyer, 2009; Rodionov et al., 2016). The divergence of the ITS region is usually correlated with the direction and rate of morphological speciation (Shneyer, 2009; Song et al., 2012; Rodionov et al., 2016).

Species of the genus Nitraria L. (Nitrariaceae) are a good object for studying the mechanisms of divergence, due not only to the variability of morphological features, but also to their ancient origin. Most of these species are morphologically poorly differentiated. Phenotypically different variants are often accepted as separate species, intraspecific forms, or ecological races (Banaev et al., 2015; Kovtonyuk et al., 2019; Tomoshevich et al., 2019). Widespread and polymorphic species (N. schoberi L. and N. sibirica Pall.), which are most interesting to researchers, are often difficult to distinguish from each other, especially for herbarium specimens (Peshkova, 1996; Koropachinskii, 2016).

The taxonomy of siberian Nitraria species has already been tried using karyological (Muratova et al., 2013; Banaev et al., 2018), phytochemical (Banaev et al., 2015) and morphological (Banaev et al., 2017) methods, however, molecular markers have a number of undeniable advantages over them, demonstrating significant differences at the genetic level without the involvement of environmental factors. Although sequencing of DNA fragments is still an expensive method of analysis, it can be provided accurate and highly informative data on the variability of genomes.

The purpose of this study was to conduct a comparative analysis of the nucleotide variability of the ITS region and identify its significance in the taxonomy of Nitraria.

## Materials and methods

**Taxon sampling.** Research specimens were collected from various locations (19 locations N. sibirica, 12 – N. schoberi, and 2 – N. komarovii Iljin & Lava ex Bobrov) in Russia (Altay region, Novosibirsk region, Crimea, Khakassia, Tuva), Kazakhstan, Tajikistan in 2011–2017 (Table 1). Herbarium specimens are stored in the Central Siberian Botanical Garden of the Siberian Branch of the Russian Academy of Sciences (Herbarium of the laboratory of dendrology, NSK Collection, Digital Herbarium CSBG SB RAS (http://herb.csbg.nsc.ru:8081).

**Table 1.**
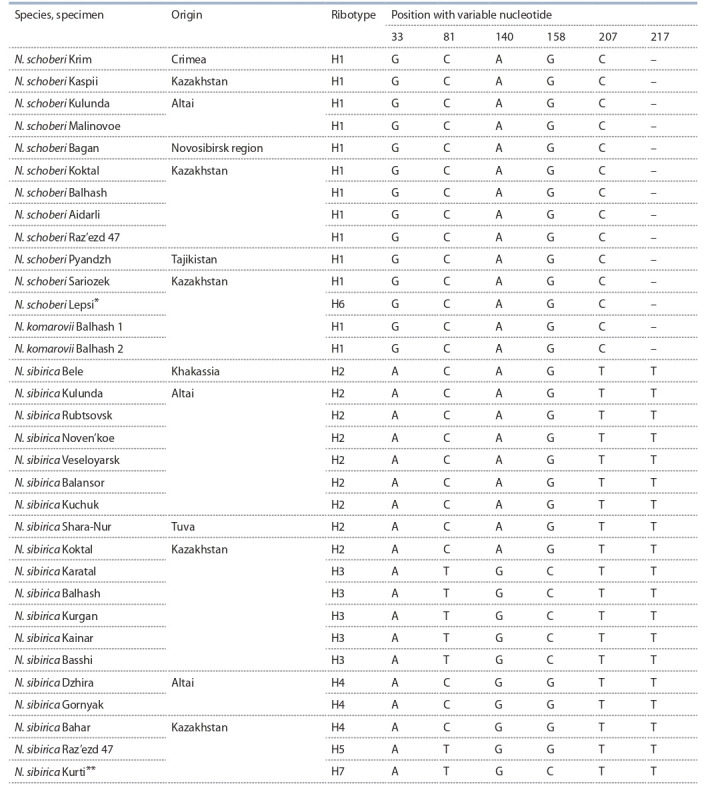
Single-nucleotide polymorphisms in ITS2 in Nitraria species Note. * The sample has a singleton at position 71; ** the sample has a singleton at position 201. A dash (–) is a single-nucleotide deletion.

**DNA extraction, PCR amplification, and sequencing.** Total genomic DNA was extracted from silica-dried leaf tissue using standard methods (CTAB) (Doyle J.J., Doyle J.L., 1990). The concentration and amount of DNA were evaluated in 0.8 % agarose gel, as well as on a spectrophotometer (NanoPhotometer P-Class, P-360, Implen).

The ITS sequences were amplified with primers ITS6 (5′-tcgtaacaaggtttccgtaggtga-3′) and ITS9 (5′-ccgcttatt gatatgcttaaac-3′), designed for East Asian species of the tribe Spiraeeae (Potter et al., 2007) and made in company Eurogen (Moscow). A ready-made set of reagents was used for PCR (GenePak® PCR Core, Laboratory Izogen, Moscow). The PCR cycle consisted of 5 min at 95 °С, 30 cycles of 1 min at 94 °С, 50 s at 58 °С, 1 min at 72 °С, and 5 min at 72 °С. PCR products were examined by electrophoresis on 1.5 % agarose gel, and the DNA fragments were subsequently extracted from the ethidium bromide-stained gel and purified using Diatom DNA Elution Kit (Laboratory Izogen, Moscow). ITS fragments were sequenced in the forward and reverse directions (Eurogen, Moscow).

**Nucleotide sequence and phylogenetic analyses.** The nucleotide sequences of the ITS region of all Nitraria specimens were aligned pairwise with BioEdit v.7.1.9 (Hall, 1999). Multiple alignments were performed in the ClustalW2 program with subsequent verification of ambiguous positions on chromatograms and manual editing. The nucleotide composition in the ITS region, the analysis of aligned sequences, selection of the nucleotide substitutions model, and evolutionary constructions were generated using MEGA X software (Kumar et al., 2018) based on the Bayesian information criterion BIC by jModelTest v.2.1.7 (Guindon, Gascuel, 2003; Darriba et al., 2012). The evolutionary distances were obtained by the Maximum Likelihood analytical method (ML) using the 3-parameter Tamura model (Tamura, 1992). Branch support was

estimated with 1000 bootstrap replicates in ML analyses (Felsenstein, 1985). Evolutionary constructions are also performed using MrBayes (Bayesian inference, BI) version 3.2.6 (Ronquist, Huelsenbeck, 2003; Ronquist et al., 2012) based on the substitution model – GTR (General Time Reversible) with a gamma distribution to approximate the rate of nucleotide replacement. The Markov chain Monte Carlo (MCMC) algorithm was set to run four chains simultaneously for ten million generations with a sampling of trees every 1000 generations. BI trees were visualized in FigTree version 1.4.3. Peganum harmala L. was used as the outgroup (GenBank NCBI: KX282320), closely related to the genus Nitraria. The boundaries of the ITS2 spacer are determined by comparing the ITS sequences obtained with the same fragments deposited in GenBank NCBI (N. schoberi: KP087771.1; N. sibirica: DQ267178.1).

## Results and discussion

The dataset used in this study included 33 specimens, belonging to 3 species Nitraria – N. schoberi, N. sibirica, N. komarovii. The ITS region of Nitraria was studied to solve phylogenetic problems (Temirbayeva, Zhang, 2015); however, information about the nucleotide variability of these fragments is given for the first time by us. The total of 577 bp of the rDNA ITS region (ITS1-5.8S-ITS2) was composed of 558 conservative sites, 17 – potentially parsimony informative sites (all of them are single-nucleotide substitutions/polymorphisms) and 2 singletons.

Eleven single-nucleotide substitutions, which are transitions (C/T), were detected in the intergenic spacer ITS1. The gene 5.8S consisted of 157 bp and was conservative, as expected. The intergenic spacer ITS2 contained 273–274 bp and was more conservative than ITS1. The ITS2 dataset comprised 5 parsimony informative sites (4 transitions: 2 – C/T, 2 – G/A; one transversion: G/C), one single-nucleotide deletion/insertion (T/–), 2 singletons (see Table 1). The GC content of the ITS region was 61.5 % and ranged from 59.2 to 62.7 % (Table 2). The GC content was lower in N. sibirica (59.2 %), than in N. schoberi and N. komarovii (62.7 %).

**Table 2.**
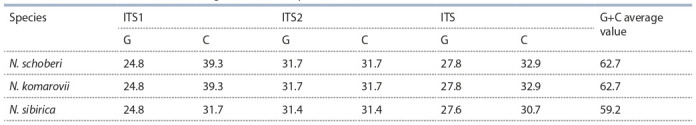
The GC content (%) of the ITS region in Nitraria species

All the transitions in ITS1 clearly separated N. sibirica from the species N. schoberi and N. komarovii, while no differences were found between N. schoberi and N. komarovii, and no intraspecific polymorphism was observed in this part of the studied samples genome. In the ITS2 spacer, both species-specific polymorphisms that distinguish N. sibirica from the other two species were identified, as well as intraspecific variability of N. sibirica specimens.

It is known that the ITS2 spacer is offered as a DNA barcode for plant identification (Feng et al., 2016). Compared to the full-size region of ITS, the shorter fragment of ITS2 is a suitable molecular marker that distinguished the studied species, due to low interspecific variability and at the same time expressed intraspecific variability. The results showed that the intergenic spacer ITS2 was different between N. sibirica and N. schoberi, as well as between N. sibirica and N. komarovii by 6 positions (5 single-nucleotide polymorphisms and one single-nucleotide deletion/ insertion). For the ITS sequence data set, p-distance value was 0.092 between N. schoberi и N. sibirica, what is comparable to well-distinguished species. For example, the average p-distance value calculated for ITS data set Dendrobium species and sections ranged from 0.069 to 0.112 (Srikulnath et al., 2015). Interspecific differences in the complex of phenolic compounds were also identified for N. sibirica and N. schoberi (Banaev et al., 2015) and species specificity of metric and qualitative morphological features was shown (Banaev et al., 2017).

Phylogenetic trees constructed separately for the ITS1 and ITS2 spacers, as well as separately for the full-size ITS region and the ITS2 spacer, were congruent. The ML and BI phylogenetic trees have branches with high bootstraps and are consistent with the morphology and taxonomy of the Nitraria genus. At the same time, during the study of the phylogeny of the Nitraria based on the analysis of combined data of ITS sequences and fragments of chloroplast DNA (6 genes) (Temirbayeva, Zhang, 2015) the species N. schoberi, N. sibirica and N. komarovii were grouped in one clade together with the Australian species N. billardieri DC., while N. komarovii, N. billardieri and N. sibirica were more closely located.

**Fig. 1.**
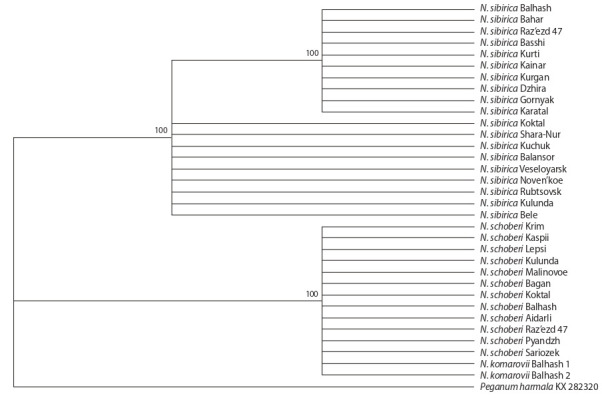
Phylogenetic tree based on a comparison of the sequences of the ITS2 spacer for Nitraria samples using the maximum likelihood method. The branches indicate the name of the species and the place of collection of the investigated sample.

**Fig. 2.**
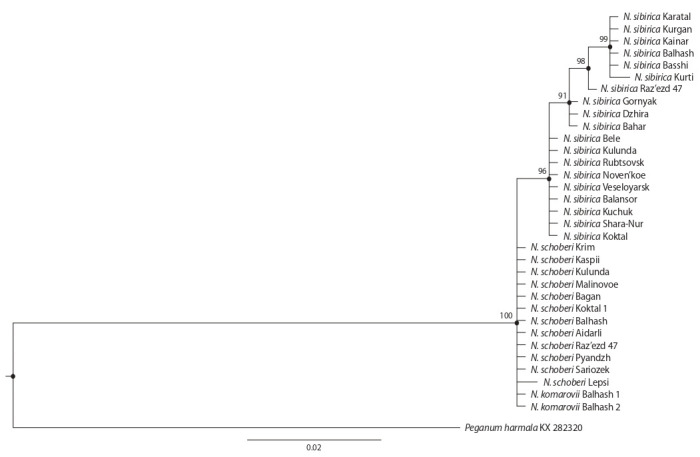
Phylogenetic tree based on a comparison of the sequences of the ITS2 spacer for the Nitraria samples using the Bayesian (BI) method. The branches indicate the name of the species and the place of collection of the investigated sample.

A comparison of the topologies of ML and BI trees (Fig. 1, 2) showed the similarity of N. schoberi and N. komarovii and the complex intraspecific differentiation of N. sibirica.

The species N. schoberi and N. komarovii with the same ITS sequences formed one separate clade and, accordingly, one ribotype – H1 (see Table 1). The exception was a sample of N. schoberi Lepsi from Kazakhstan, characterized by the presence of a singleton in position 71 of the spacer ITS2 (see Table 1).

The specimens of N. sibirica were grouped into two subclades on the ML phylogenetic tree (see Fig. 1), and three subclades – on the BI tree (see Fig. 2). One of the ribotypes (H3) of N. sibirica differed in six single-nucleotide substitutions from the N. schoberi and N. komarovii, which indicates an independent taxonomic rank of these populations. The average intergroup genetic distance, which was 0.024 and was the same for both the H1/H2 and H1/ H4 groups, confirmed the same. Ribotypes H2, H3, H4 belonging to N. sibirica differed by 1–3 single-nucleotide substitutions. Each of the H5, H6, and H7 ribotypes had one-point mutation (substitution).

**Fig. 3.**
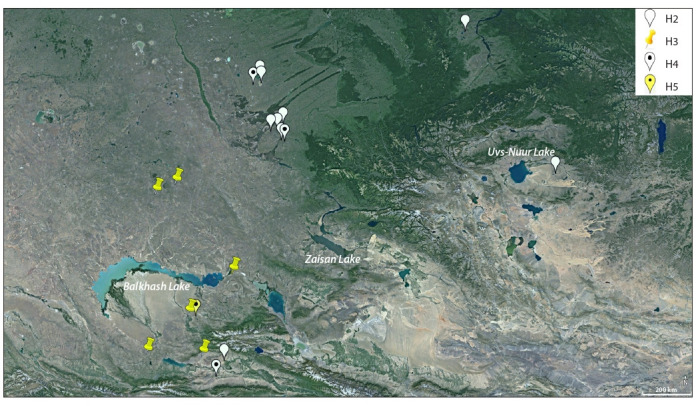
Spatial distribution of ITS ribotypes of N. sibirica (H2, H3, H4, H5).

As a result of our research on the intraspecific differentiation of N. sibirica the samples were divided into two main ribotypes: the main Siberian sibirica-ribotype (Н2) and the main Kazakh sibirica-ribotype (Н3) (Fig. 3).

The H2 ribotype was common in the Siberian populations of N. sibirica – the Altai territory (Kulundin steppe), Khakassia, and Tuva. The H4 ribotype, which differed in one single-nucleotide substitution from the main Siberian sibirica-ribotype, was also common in populations growing mainly in Kulunda, excluding two populations of N. sibirica from South-Eastern Kazakhstan on the border with China – Koktal and Bahar, where the Siberian sibiricaribotype (H2) and the H4 ribotype close to it were found.

The main Kazakh sibirica-ribotype (H3) was distributed in the Ili-Balkhash region (Ili, Karatal, Ayaguz river basins) and the Kazakh shallow-water area. Ribotypes H5 and H7, close to the H3 ribotype, were also found in the distribution region of the main Kazakh sibirica-ribotype.

We noted significant inter-population differences and taxonomic heterogeneity of N. sibirica due to geographical distribution of N. sibirica ribotypes, as well as significant differences between the main Siberian and main Kazakh sibirica-ribotypes (3 single-nucleotide substitutions). Most likely, the processes of homogenization of nuclear ribosomal DNA of N. sibirica samples, whose origin is associated with hybridization and speciation (Rauscher et al., 2003; Xu et al., 2017; Efimova et al., 2019), are currently continuing. Previously, it was shown that the populations of N. sibirica were heterogeneous and differentiated into separate groups according to ecological and geographical features and the gradient of height above sea level by a complex of phenolic compounds (Banaev et al., 2015).

## Conclusion

The obtained results of comparative analysis of the nucleotide variability of the ITS region demonstrated the reliability of the ITS2 spacer as a molecular genetic marker in the identification of Nitraria species. In the case of complex morphological identification of Nitraria samples, a genetic analysis of the variability of the short ITS2 spacer could be sufficient. However, it should be noted that the ITS region may not always fully resolve all taxonomic issues. Thus, in our study, the species N. schoberi and N. komarovii had identical its sequences. In addition, difficulties in interpreting the obtained sequence data set could be related to multiple copies of ITS, which are paralogs or orthologs. Answers to further questions related to the taxonomy and evolution of Nitraria species can be obtained by identifying these homologues, cloning its fragments, and using additional genetic markers of the chloroplast genome. In addition, the identified species-specific genetic polymorphisms in the ITS region in the studied Nitraria species will allow further selection of restriction enzymes and thus simplify and reduce the cost of obtaining patterns of genetic variability of closely related taxa Nitraria.

## Conflict of interest

The authors declare no conflict of interest.
